# Correction: green synthesis of silver nanoparticles using plant extracts and their antimicrobial activities: a review of recent literature

**DOI:** 10.1039/d2ra90055f

**Published:** 2022-05-31

**Authors:** Chhangte Vanlalveni, Samuel Lallianrawna, Aayushi Biswas, Manickam Selvaraj, Bishwajit Changmai, Samuel Lalthazuala Rokhum

**Affiliations:** Department of Botany, Mizoram University Tanhril Aizawl Mizoram 796001 India; Department of Chemistry, Govt. Zirtiri Residential Science College Aizawl 796001 Mizoram India; Department of Chemistry, National Institute of Technology Silchar Silchar 788010 India rokhum@che.nits.ac.in lr512@cam.ac.uk bishwajit_rs@che.nits.ac.in; Department of Chemistry, Faculty of Science, King Khalid University Abha 61413 Saudi Arabia; Department of Chemistry, University of Cambridge Lensfield Road Cambridge CB2 1EW UK

## Abstract

Correction for ‘green synthesis of silver nanoparticles using plant extracts and their antimicrobial activities: a review of recent literature’ by Chhangte Vanlalveni *et al.*, *RSC Adv.*, 2021, **11**, 2804–2837, https://doi.org/10.1039/D0RA09941D.

The authors regret that the name of one of the authors (Aayushi Biswas) was shown incorrectly in the original article. The corrected author’s name is as shown above.

The Royal Society of Chemistry apologises for these errors and any consequent inconvenience to authors and readers.

## Supplementary Material

